# The Effects of an Exposure-Based Mobile App on Symptoms of Posttraumatic Stress Disorder in Veterans: Pilot Randomized Controlled Trial

**DOI:** 10.2196/38951

**Published:** 2022-11-04

**Authors:** Carmen McLean, C Adrian Davis, Madeleine Miller, Josef Ruzek, Eric Neri

**Affiliations:** 1 National Center for Posttraumatic Stress Disorder, Dissemination and Training Division Department of Veterans Affairs Menlo Park, CA United States; 2 Department of Psychiatry and Behavioral Sciences Stanford University Palo Alto, CA United States; 3 Department of Psychology University of Colorado Colorado Springs, CO United States

**Keywords:** posttraumatic stress disorder, veteran’s health, exposure therapy, cognitive behavioral therapy, mHealth, mobile apps, self-management

## Abstract

**Background:**

Barriers to accessing in-person care can prevent veterans with posttraumatic stress disorder (PTSD) from receiving trauma-focused treatments such as exposure therapy. Mobile apps may help to address unmet need for services by offering tools for users to self-manage PTSD symptoms. Renew is a mobile mental health app that focuses on exposure therapy and incorporates a social support function designed to promote user engagement.

**Objective:**

We examined the preliminary efficacy of Renew with and without support from a research staff member compared with waitlist among 93 veterans with clinically significant PTSD symptoms. We also examined the impact of study staff support on participant engagement with the app.

**Methods:**

In a pilot randomized controlled trial, we compared Renew with and without support from a research staff member (active use condition) with waitlist (delayed use condition) over 6 weeks. Participants were recruited through online advertisements. The Posttraumatic Stress Disorder Checklist for Diagnostic and Statistical Manual of Mental Disorders, fifth edition (DSM-5) was used to measure PTSD symptoms at pre, post, and 6-week follow-up. Usage data were collected to assess engagement with Renew.

**Results:**

Results indicated a small effect size (d=–0.39) favoring those in the active use conditions relative to the delayed use condition, but the between-group difference was not significant (*P*=.29). There were no differences on indices of app engagement between the 2 active use conditions. Exploratory analyses found that the number of support persons users added to the app, but not the number of support messages received, was positively correlated with app engagement.

**Conclusions:**

Findings suggest Renew may hold promise as a self-management tool to reduce PTSD symptoms in veterans. Involving friends and family in mobile mental health apps may help bolster engagement with no additional cost to public health systems.

**Trial Registration:**

ClinicalTrials.gov NCT04155736; https://clinicaltrials.gov/ct2/show/NCT04155736

## Introduction

Posttraumatic stress disorder (PTSD) is an often chronic and debilitating condition associated with psychiatric and physical comorbidities, functional impairments [1], and elevated health care utilization [2]. PTSD affects a significant minority (8%) of the general US population. An estimated 14% of veterans returning from Iraq and Afghanistan meet the diagnostic criteria for PTSD [3] and an additional 7.6% experience clinically significant symptoms that warrant intervention but do not meet diagnostic criteria [4].

Trauma-focused psychotherapy (ie, therapy that addresses traumatic memories and trauma-related appraisals and includes exposure or cognitive restructuring as a primary therapeutic component) is recommended as the first-line treatment for veterans with PTSD [5]. However, not all veterans with clinically significant symptoms of PTSD are willing or able to access this kind of care. A lack of trained providers, logistical barriers (eg, travel for in-person care), and concerns about stigmatization may prevent veterans from receiving these gold-standard interventions [6]. Mobile mental health apps that include trauma-focused techniques may be able to overcome these barriers and increase the reach of such effective intervention approaches.

Mobile mental health apps have proliferated in recent years and are accessible to the 97% of US adults who now own smartphones [7]. They can provide anonymous, on-demand intervention 24/7 at very low cost. However, despite the growing number of mobile mental health apps, apps targeting PTSD are less common, and more evidence testing the efficacy of apps addressing PTSD symptoms is needed [8,9].

The most well-supported app for PTSD is PTSD Coach, a self-management tool designed to help users cope with symptoms of PTSD. PTSD Coach has been evaluated in 3 randomized controlled trials (RCTs) to date. A small initial pilot compared 8 weeks of PTSD Coach use with and without clinician coaching in a small sample of veterans and found that both groups experienced reductions in PTSD symptoms, with a medium effect size (*d*=0.54) favoring coaching but no statistically significant group differences (*P*=.02; N=20; [10]). The next pilot trial compared 1 month of app use with waitlist among community members with elevated PTSD symptoms (N=49) and found a small effect favoring PTSD Coach (*d*=0.25) that did not reach statistical significance (*P*≥.05) [11]. The third trial examined 12 weeks of app use among community members with elevated PTSD symptoms (N=120) and found that participants who received PTSD Coach showed significantly greater reductions in symptoms compared with waitlist, (*P*=.035) with improvements maintained at the 3-month follow-up (*P*=.113) [12]. However, differences between the PTSD Coach group and waitlist were not significant (*P*≥.05) at the postintervention period.

Aside from studies of PTSD Coach, another RCT provided service members with moderate PTSD symptoms (N=144) access to several mobile apps that included psychoeducation, social engagement, and relaxation exercises. Participants then received either a 6-week cognitive behavioral program designed to promote app use or 6 weeks of daily inspirational SMS text messages [13]. There were no differences between groups; both groups showed reductions in PTSD that were maintained at 3-month follow up, but deteriorated slightly 6-12 months later.

In summary, mobile apps may be useful for individuals with PTSD symptoms, whether used alone or with coaching, but current evidence is relatively weak and limited to only a few RCTs. Currently, PTSD Coach is the only app for PTSD with evidence to support its use as a stand-alone self-management tool. However, because PTSD Coach does not directly address thoughts, feelings, or memories of the traumatic event(s) through exposure or cognitive restructuring, it is not a trauma-focused intervention.

The most studied trauma-focused treatment for PTSD is exposure therapy. Exposure therapy involves approaching the traumatic memory (imaginal exposure) or trauma-related situations and stimuli in real life (in vivo exposure). Extensive evidence supports exposure therapy’s efficacy for treating PTSD in a range of trauma survivors [14]. It is also underutilized by mental health providers because many providers believe exposure therapy is more difficult for patients to tolerate than other evidence-based treatments [15,16], although this perception is not supported by empirical evidence [17,18]. Exposure has been implemented successfully in web-based interventions, including cognitive behavioral programs with written imaginal exposure [19-22], in vivo exposure [23], and both [24]. However, despite promising findings from web-based interventions, a recent review found that fewer than 10% of all mobile apps targeting PTSD include any exposure component [8], and to our knowledge, no studies have examined the efficacy of PTSD self-management apps that use exposure techniques. An important question, then, is whether apps that include exposure therapy exercises can reduce PTSD symptoms.

Sustained user engagement is a key challenge with all mobile mental health apps [25]. Most apps show a steep reduction in usage after download, with only 1%-5% of users continuing to use the app after 30 days [26]. Human feedback can provide guidance, emotional support, and encouragement to increase accountability and engagement with app-based interventions [27-29]. Studies suggest that telephone support from trained peer coaches [30] or clinicians [31] may improve engagement with PTSD Coach. However, use of trained support personnel limits scalability of such interventions due to challenges associated with training, supervising, and monitoring coaches. Thus, another unanswered question is whether support from a friend or a family member can provide the same benefit.

To address these research gaps, we developed Renew, an exposure-based self-management mobile app for PTSD that includes a peer support component. We recently demonstrated the feasibility and acceptability of Renew in a small study of 18 adults who had experienced a traumatic event [32]. Qualitative data from this study suggested that users who were motivated to work independently to manage their PTSD felt the app would be helpful in reducing symptoms. Building on this initial study, this RCT aimed to assess the preliminary efficacy of Renew in reducing PTSD symptoms for veterans with PTSD, as well as examine the impact of the support component on app engagement. We hypothesized that veterans who used Renew for 6 weeks (*active use condition*) would show greater reductions in PTSD symptoms relative to the *delayed use condition*, and that those who were assigned to receive support from project staff (*support condition*) would show greater app engagement than those who were not provided with a support person (*no support condition*). We also explored relationships between indices of support (number of peer support persons added to the app and number of support messages received from peer supporters) and indices of app engagement (time spent using the app, time spent using exposure components of the app, and number of points gained in the app, as a proxy for activity completion).

## Methods

### Description of the Renew App

Renew is a self-management app for symptoms of PTSD. Renew is unique from PTSD Coach in that it was designed to treat PTSD symptoms, whereas PTSD Coach was designed to help people manage and reduce acute experiences of distress. Renew was developed by Vertical Inc. and is owned by the Department of Veterans Affairs. An initial build of the app was revised using feedback from volunteers who had experienced a traumatic event during a small feasibility and usability study of Renew [32].

The app includes 2 primary exposure components, namely, *process* and *approach*. Both include a rationale, detailed instructions, and examples, with prompts for users to provide a 0-100 subjective unit of distress (using the Subjective Units of Distress Scale [SUDS]) rating before and after each activity. *Process* guides users through imaginal exposure (ie, approaching trauma memories through imagination) using a series of writing prompts. In this activity, users describe their worst traumatic event for at least 20 minutes, using either their phone keyboard or the talk-to-text function. *Approach* guides users through in vivo exposure by identifying real-life situations they have been avoiding due to their trauma and building a hierarchy of situations for them to approach.

**Figure 1:**
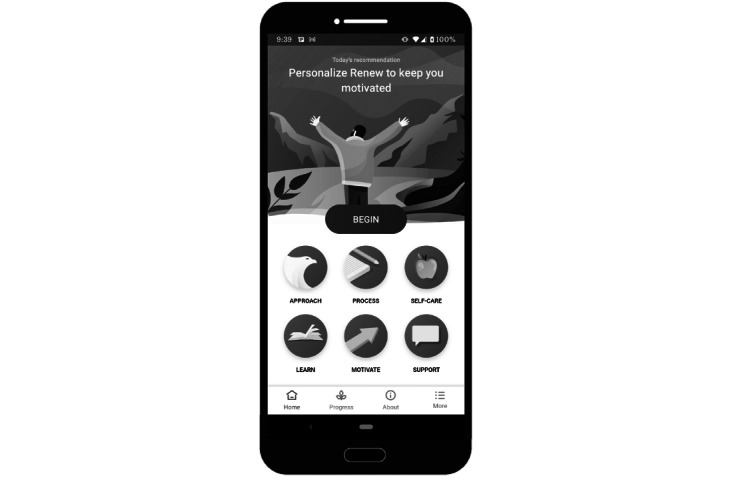
Sample screenshot of the Renew app home screen. The home screen displays the 6 primary components of the app. During onboarding, the “Begin” button takes users to a short, animated video that outlines the features of Renew and the rationale behind exposure as an approach to managing posttraumatic stress.

**Figure 2:**
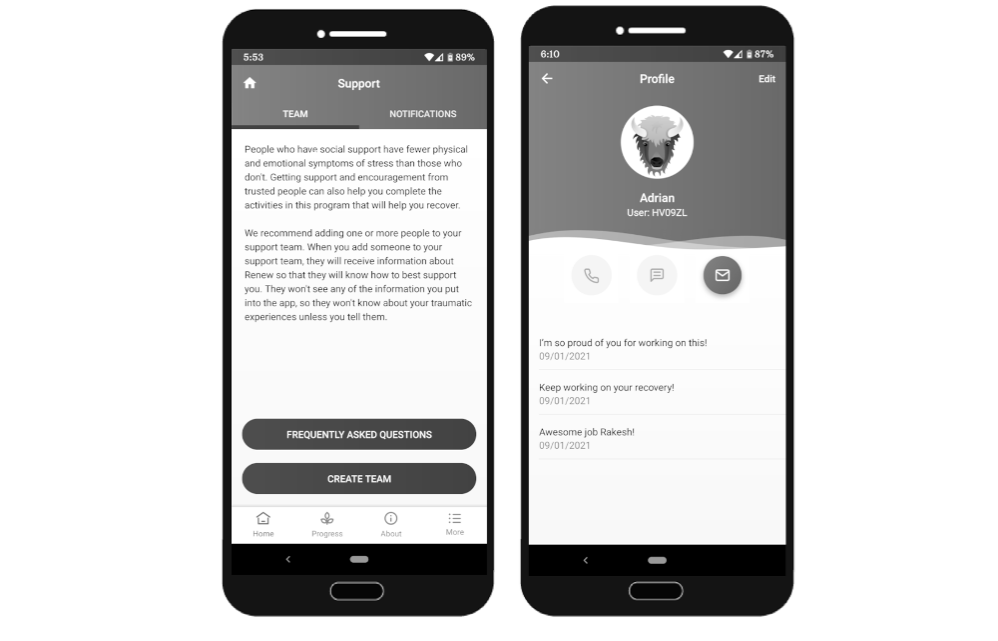
Sample screenshots of the Renew app—*support*. The image on the left displays some of the explanation of the *support* feature in Renew. The image on the right displays an example of the messages a user might see from one of their *support* team members in response to usage notifications.

**Figure 3:**
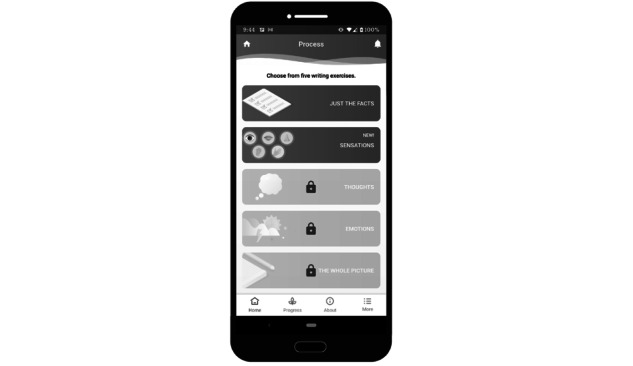
Sample screenshot of the Renew app—*process*. This image shows the writing prompts available to users. Prompts are unlocked as the user progresses in the program.

**Figure 4:**
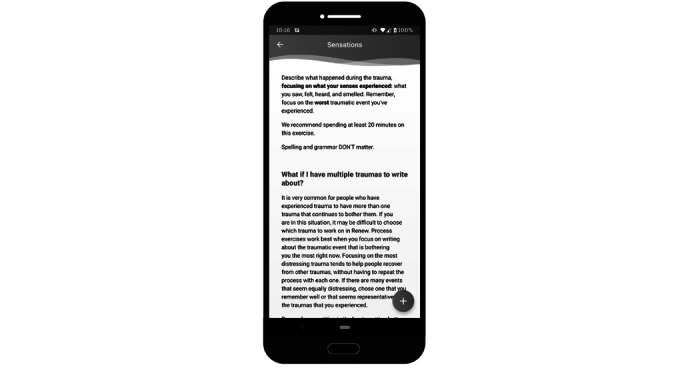
Sample screenshot of the Renew app—*process*. This image shows a portion of the instructions shown to users after clicking on the writing prompt sections.

**Figure 5:**
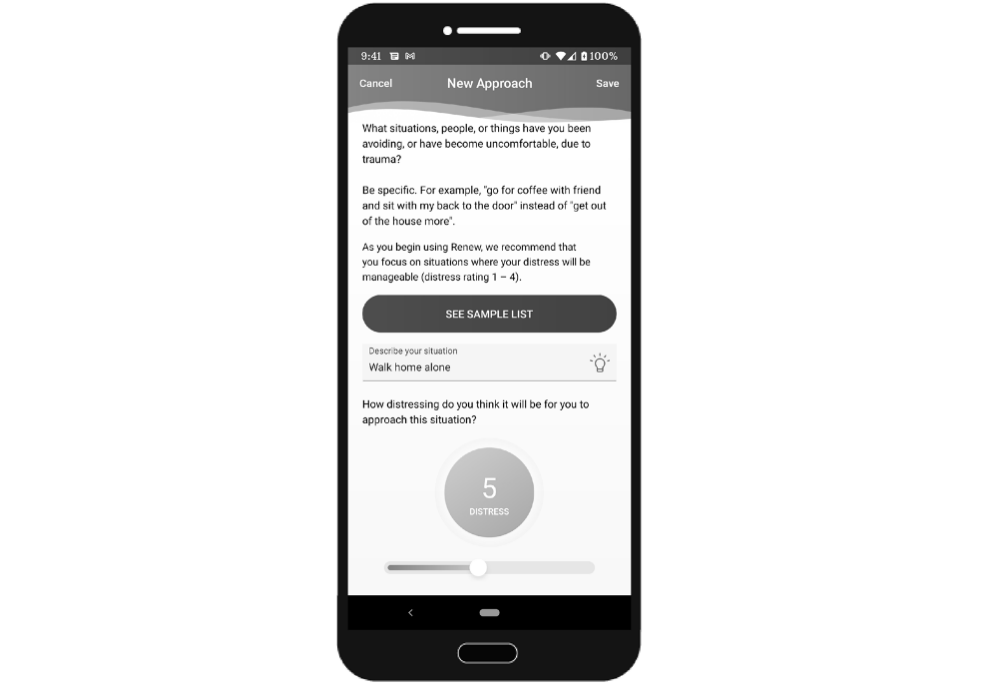
Sample screenshot of the Renew app—*approach*. This image shows some of the instructions shown to users in the *approach* section of the app.

**Figure 6:**
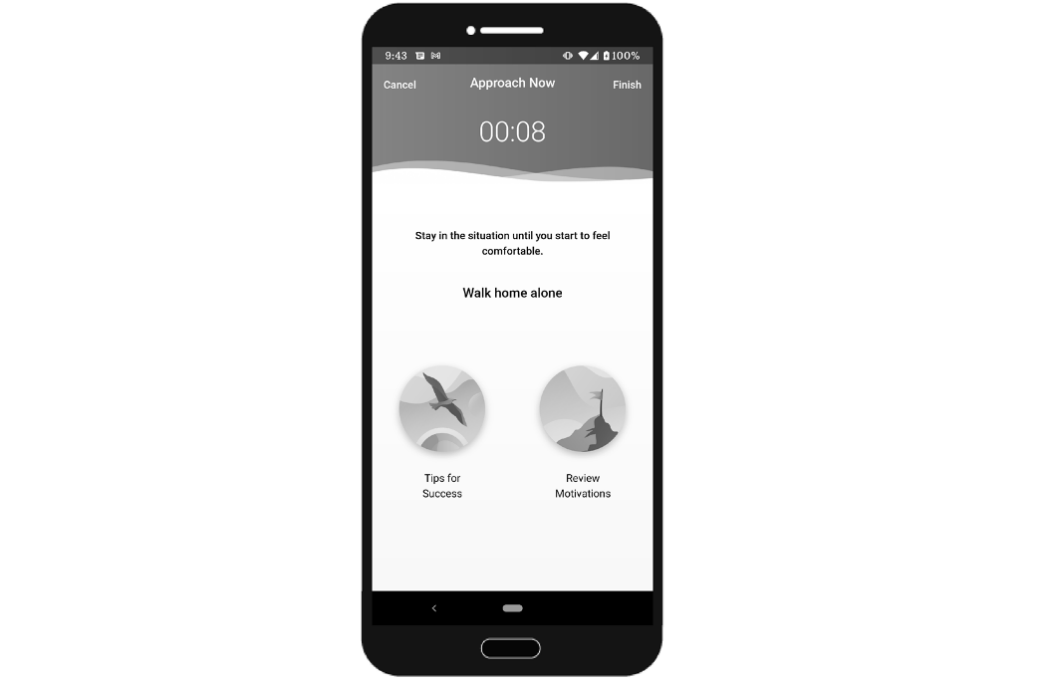
Sample screenshot of the Renew app—*approach*. This image displays the screen shown to users who have initiated an *approach* activity. The activity is timed, and they have access to a list of tips as well as the content in the motivation section.

A *learn* section includes psychoeducation about common reactions to trauma, the development of PTSD symptoms, the role of avoidance in maintaining symptoms, and the science behind exposure therapy techniques. The *self-care* section allows users to identify and schedule self-care activities that promote relaxation, physical activity, and social engagement. *Support* allows users to invite trusted friends or family members to be part of their support team in Renew. Support persons are invited to download “Renew Support” (available on Apple and Android smartphones), which provides psychoeducation about PTSD symptoms and information on how to support someone who is working on managing PTSD symptoms. Support team members receive notifications when the primary user earns points or achieves a new level and are instructed to send an encouraging message through the app’s 1-way message system. *Motivate* allows users to personalize the app by selecting quotes, videos, and images that motivate them to work on managing their PTSD symptoms. Users may add their own or select from Renew’s built-in options. In *progress,* users can complete a 5-item “symptom tracker” assessing coping self-efficacy, depression, and PTSD symptoms. Users can view the results of these assessments, as well as the SUDS data from *process* and *approach*, over time in a graph view (Figures 1-6). Renew prompts users to complete assessments every 2 weeks.

In addition to the main sections, Renew includes elements of gamification: users earn points for completing activities in the app and can level up to unlock new features and activities. Crisis resources are also listed in the app. Users can turn on daily notifications and receive reminders to use Renew.

### Participants

**Figure 7:**
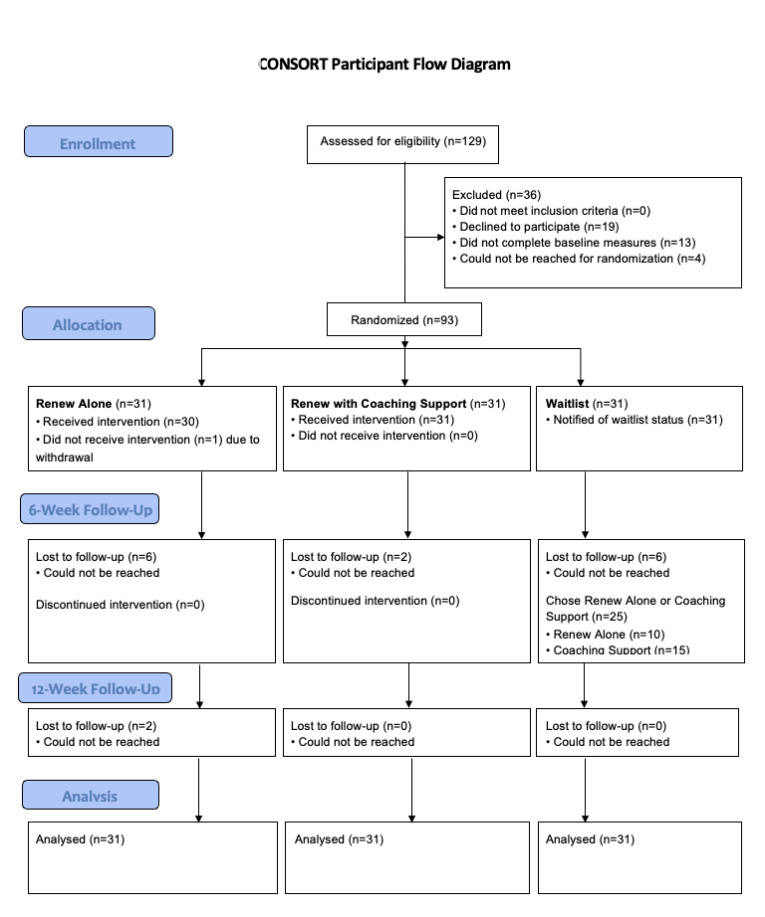
CONSORT (Consolidated Standards of Reporting Trials) participant flow diagram.

Participants were recruited via online advertisements from across the United States (Figure 7 and Multimedia Appendix 1). Inclusion criteria were age 18 years or older, veteran status, exposure to a criterion A trauma, a PTSD symptom severity score of 31 or greater [33] on the PTSD Checklist for the Diagnostic and Statistical Manual of Mental Disorders, fifth edition (DSM-5) [34], and access to an Android smartphone.

### Measures

#### Demographics

This is a 14-item baseline measure of demographic information, including race, ethnicity, age, gender, and military service.

#### Posttraumatic Stress Disorder Checklist for DSM-5

The Posttraumatic Stress Disorder Checklist for DSM-5 (PCL-5) [34] is a 20-item self-report measure of PTSD symptoms as defined by the Diagnostic and Statistical Manual of Mental Disorders [35]. The PCL-5 has high internal consistency (Cronbach α=.91–.95 [36]) and strong test-retest reliability [37,38]. The PCL-5 was delivered as an online survey. The psychometric properties of the PCL-5 have been previously tested when used in an online format [38].

#### Renew Usage Data

A research dashboard collected information on app usage, including button presses, activities completed, length of time users engaged with each app section, and number of characters entered into free text fields of the app. No identifying information was collected.

To measure user engagement with the Renew app, the following variables were included in our analyses: time spent using the app, time spent completing exposure activities, and the number of points a user gained for completing activities in the app. All time variables were measured in seconds, but are presented in minutes in this paper for ease of interpretation. “Exposure activities” included both in vivo and written exposure tasks (activities in the approach and process sections of the Renew app). Users gained points for completing activities in the app, with users gaining more points for completing exposure activities than for nonexposure activities. The number of points a user gained over the 6-week period serves as a nontemporal metric of engagement and a proxy for the number and type of activities users completed over the app-use period. All usage variables reported in this paper describe app usage during the 6-week active use period for all participants, regardless of study condition.

### Study Conditions

#### Renew Alone

Participants received a 30-minute orientation to Renew and were invited to use the app as much as they wanted for 6 weeks. Participants were allowed, but not instructed, to invite peer support persons who could send 1-way messages of encouragement through the Renew app.

#### Renew With Study Staff Support

Participants received a 30-minute orientation to Renew and were instructed to add a study research assistant (RA) to their support team. Participants were allowed to invite additional peer support persons as they wished. Support team members are notified when the participant levels up or earns points. The assigned study RA responded to all notifications with reinforcing messages (ie, “Great job staying focused on recovery!”) or when the participant had not earned points or gained a level in 7 or more days (ie, “Hey, it’s been a while since you’ve used Renew. Do you have time to focus on recovery today?”). Participants were instructed to use the app as much as much as they wanted for 6 weeks.

#### Delayed Use

Participants were informed that an RA would reach out to them in 6 weeks to schedule the app orientation session with them and that they could elect to receive study staff support or not.

### Procedures

Potential participants completed an online screening assessing study enrollment criteria. Eligible participants were then emailed the informed consent information sheet and met with a study RA by phone to complete the informed consent process (see Multimedia Appendix 2). After completing a baseline assessment, consented participants were randomized by the study RA and informed of their condition assignment over the phone, at which point participants and researchers were unblinded to study condition. Randomization was done by a study RA using an online random block generator allocating participants to the 3 groups using a 1:1:1 ratio. Participants assigned to either of the 2 active treatment conditions were guided through downloading Renew and given a special code to allow them access to Renew’s content during a 30-minute in-depth orientation to the app delivered by a study RA.

After the 6-week app-use phase, participants completed a postuse online survey and a brief telephone interview. All surveys were sent to the participants’ emails via REDCap, a secure online data collection tool that enables users to design and administer questionnaires. Interviews were audio-recorded and transcribed by study RAs. Participants in the active use conditions completed a follow-up assessment online 6 weeks after the postuse assessment (ie, week 12). Participants in the delayed use condition only completed pre- and postuse surveys. Participants were compensated with an online gift card in the amount of US $100 after study completion. Study data were collected from July 18, 2020, to February 3, 2021.

### Statistical Analysis

Our first hypothesis was that veterans in either treatment condition would show greater reductions in PTSD symptoms relative to delayed use. We also hypothesized that veterans who were assigned a support team member would engage with Renew more and show greater reductions in PTSD symptoms than those who were not assigned a support team member. In addition, we explored the relationship between indices of support and indices of app engagement.

The primary hypothesis was tested using the MIXED procedure [39] in SAS 9.4 (SAS Institute) [40] to fit a mixed analysis of variance (ANOVA) model. Restricted maximum likelihood estimation was used to conduct the analysis following the intention-to-treat principle and allow for missing data. For the equally spaced time points, which included the 6 weeks from baseline to posttreatment and the 6 weeks from posttreatment to follow-up, the model assumed an autoregressive error covariance structure. The model included time and treatment group as factors with the interaction term testing group differences in PCL-5 change or slope, which was the primary outcome of interest.

For analyses of indices of support, the variables of interest were characterized by skew distributions where nonparametric tests are more appropriate: for the secondary hypothesis comparing indices of support between the 2 active use conditions, we used the Mann-Whitney-Wilcoxon test and area under the receiver operating characteristic curve effect size [41]. For the exploratory analysis of associations with indices of support, we used the Spearman’s rank correlation coefficient.

### Ethics Approval

This study was approved by the Stanford Institutional Review Board (IRB-52829).

## Results

After trial commencement, participants detected a bug that caused the app to crash when the *approach* feature was opened. This was reported by 11 participants before it was corrected halfway through the study period.

Participant demographics are reported in Table 1. Participants were predominately women (64/93, 69%) and reported a mean age of 49 (SD 9.3) years.

**Figure 8:**
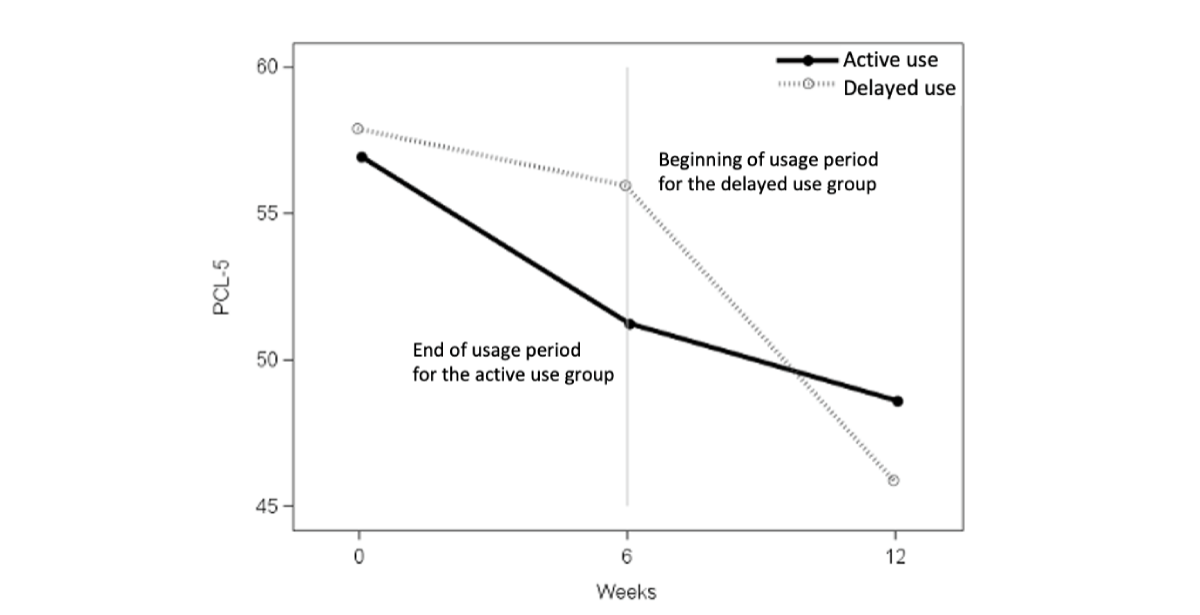
PCL-5 score comparison—active use versus delayed use. PCL-5: Posttraumatic Stress Disorder Checklist for DSM-5.

The mixed model ANOVA estimates showed a larger PCL-5 decrease during the 6-week period for the active use group compared with the delayed use group (–6.14 vs –1.84; Figure 8). This difference was not significant (*P*=.29), with a small effect size (*d*=–0.39) [42]. Using the model estimates, the within-group PCL-5 change from baseline to posttreatment was significant for the combined active use participants (–6.14, 95% CI –10.66 to –1.62; *P*=.008), but not for the delayed use group. During the usage period from 6 to 12 weeks for the delayed use group, the within-group PCL-5 change was also significant (–10.1, 95% CI –15.7 to –4.44; *P*<.001).

**Table 1:** Participant demographics (N=93).

Demographics		Values, n (%)	
**Gender^a^**			
	Men	29 (31)	
	Women	64 (69)	
**Race^b^**			
	White or European American	59 (63)	
	Black or African American	22 (24)	
	Another racial identity	11 (12)	
	American Indian or Alaska Native	5 (5)	
	Asian or Asian American	3 (3)	
	Middle Eastern/North African	1 (1)	
**Ethnicity**			
	Not Hispanic or Latinx	83 (89)	
	Hispanic or Latinx	10 (11)	
**Relationship status**			
	Married/partnered	33 (35)	
	Single, not in a relationship	23 (25)	
	Separated/divorced	20 (22)	
	Single, in a relationship	15 (16)	
	Widowed	2 (2)	
**Education**			
	Some high school	1 (1)	
	Some college or a 2-year degree	47 (51)	
	Earned a 4-year degree	21 (23)	
	Postgraduate	19 (20)	
	High school or General Educational Development	5 (5)	
**Employment**			
	Not currently working for pay	35 (38)	
	Full-time work	29 (31)	
	Retired	20 (22)	
	Part-time work	9 (10)	
**Military status**			
	Veteran	81 (87)	
	Retired	11 (12)	
	National Guard	1 (1)	

^a^No participants identified as transgender, nonbinary, or another gender identity.

^b^Participants were allowed to select more than 1 race.

During the 6-week active use period, the median number of minutes spent using Renew was 104.75 (IQR 42.32-218.42). There were no differences between the support and no support groups on engagement indices (Table 2). Comparing the PCL-5 changes between the 2 active use conditions, the 0- to 6-week change in PCL-5 estimated by the mixed ANOVA model was –8.54 (95% CI –15.2 to –1.93) for the no support condition compared with –4.02 (95% CI –10.3 to 2.24) for the support condition, resulting in a small effect size difference of –0.40. This difference was not significant (*P*=.28).

**Table 2:** IQR, *P* values, and AUC^a^ for usage variables.

Variables	No support (n=30), median (IQR)	Support (n=31), median (IQR)	Mann-Whitney-Wilcoxon test, *P* value	Effect size AUC (rating)
Time^b^ in app (minutes)	102 (67-267)	122 (57-231)	.72	0.53 (null)
Time^b^ in exposure (minutes)	41 (3-113)	27 (6-106)	.91	0.49 (null)
Number of points	155 (50-410)	185 (60-510)	.52	0.55 (null)

^a^AUC: area under the receiver operating characteristic curve.

^b^All time variables were measured in seconds but are presented here in minutes for ease of interpretation.

Because participants in both the support and no support conditions were able to add their own support team members (eg, family or friends), we explored the impact of the support feature in 2 additional ways: the number of total support messages received (including study staff and peer messages; these could not be disaggregated) and the number of support team members added (excluding the study staff member). Participants in the support condition received a median of 19 messages (IQR 18-21) and participants in the no support condition received a median of 0 messages (IQR 0-0). As shown in Table 3, none of the associations were significant or met the criterion for moderate effect size rating (Spearman ρ≥0.3; *P*≥.05). However, when support is operationalized as the number of support persons added (excluding the staff person among those in the support condition), there are significant associations (*P*=.004 for time in app, *P*=.02 for time in exposure, and *P*=.005 for number of points; Table 3). with engagement variables. More support persons added was associated with greater time in app, more time in exposure, and a greater number of points.

**Table 3:** Correlation (Spearman ρ) between number of support messages/support persons and app engagement (all groups combined).

Variable	Number of messages, ρ (95% CI)	*P* value	Number of people, ρ (95% CI)	*P* value
Time in app (seconds)^a^	0.17 (–0.05 to 0.37)	.13	0.31 (0.10 to 0.49)	.004
Time in exposure (seconds)	0.08 (–0.13 to 0.29)	.45	0.26 (0.05 to 0.44)	.02
Number of points	0.21 (–0.00 to 0.40)	.05	0.30 (0.09 to 0.48)	.005

^a^Although time variables are presented elsewhere in the paper in terms of minutes for ease of interpretation, our analyses were conducted in terms of seconds, so that is how our data are presented in this table.

## Discussion

### Principal Findings

Our results provide support for the preliminary efficacy of Renew, with a small effect favoring app use compared with delayed use. The between-group difference was not significant, although veterans who used Renew reported reductions in PTSD symptoms, whereas veterans in the delayed use condition did not, until they began using Renew. PTSD symptom reduction during the active use phase was approximately 6 points on the PCL-5 for those in the active use condition and 10 points for those in the delayed use condition, which is comparable to findings from a study of PTSD Coach in a community sample of trauma survivors over a 1-month period (7 points) [11]. These findings suggest that Renew may represent another effective self-management tool to reduce PTSD symptoms.

The 6-week use period was short relative to other studies and may have underestimated the effect of Renew. The pattern of PTSD symptom change indicates some continued improvement during the follow-up period, which is consistent with research on PTSD Coach showing that symptoms declined over a 3-month use period and, to a lesser degree, a subsequent 3-month follow-up [12]. Thus, it seems warranted to examine the impact of Renew over a longer use period in future studies.

Contrary to hypothesis, we did not see greater app engagement or PTSD reduction among those in the support condition, who were assigned a study staff person as one of their support team members relative to those in the no support condition. The support condition may not have been impactful because the study staff person is not someone the veteran knows personally.

Consistent with this interpretation, the results of the exploratory analyses suggested that adding friends or family as support team members did promote engagement with Renew. The number of support persons added (ie, beyond the study staff person as required by study condition) was associated with greater overall time spent in Renew, greater time spent in exposure, and achieving a greater number of points. Potential reasons for this association could be that adding a support team member, which informs that person that the user is working to manage symptoms of posttraumatic stress, could promote self-accountability or could open the door to more direct forms of social support (eg, supportive conversations between the participants and their support person). The effect could also be related to receiving encouraging messages from the peer support person(s) through Renew. This finding has important public health implications because it suggests that incorporating peers may represent an effective approach to improve app engagement at no cost. However, it is important to keep in mind that we do not know the direction of this relationship. Participants who engaged more with Renew could have been more inclined to add support persons, or those who added more support persons could have felt more encouraged to use Renew. We did not find that the number of support messages received was related to app use, but we were not able to isolate messages from peer support team members from study staff members.

Veterans in this study were instructed to use the app as much as they wanted for 6 weeks and were oriented to and encouraged to try all components of Renew. Although those in the support condition received messages in response to their use of Renew, there was no synchronous coaching provided (eg, coaching phone calls). This allowed us to approximate what the effect of Renew may be in routine use, with no additional resources provided. However, it is possible that scaffolding to support app engagement would have resulted in greater clinical benefit. Future studies could explore the impact of Renew with coaching or as an adjunct to more traditional care (eg, including a therapist as a support team member). The Efficiency Model of Support [43] could be a relevant protocol to use in future studies of Renew to further evaluate the effectiveness of the support function.

### Limitations

Several study limitations should be considered. First, participants were veterans and therefore the findings may not generalize to other populations, including civilians. Second, most of the data collection occurred during the first wave of the COVID-19 pandemic in the United States, when movement restrictions were high and vaccines were unavailable. It is unclear how much COVID-19–related stress impacted PTSD symptoms, app engagement, or outcomes in our study sample. Other research with veterans enrolled in PTSD psychotherapy suggests that the COVID-19 pandemic worsened PTSD symptoms [44] and negatively impacted engagement with treatment [45]. It may be that COVID-19–related stressors had a similarly negative impact on PTSD symptoms and engagement with Renew. We are currently examining data on COVID-19 stressors in this sample in an effort to better understand the impact of the pandemic on the veteran’s experience using Renew. Finally, we were not able to disaggregate the number of support message from study staff and peers, limiting our ability to understand how this feature was related to engagement.

### Future Directions

Given the preliminary efficacy of Renew, future research evaluating the app is warranted and several possible directions can be considered. First, based on the pattern of change during the follow-up period and findings from PTSD Coach [11], we recommend that Renew be tested in a longer use period than 6 weeks. This would allow researchers to explore an optimal “dosage” of Renew. Second, in light of the exploratory findings on the support feature, future work may also examine ways to encourage users to develop a personal support team. Third, research examining engagement with different components of Renew may be useful to determine whether the use of particular features (eg, exposure components) is most closely associated with symptom reduction. Finally, future work may consider testing Renew as a supplement to psychotherapy or as part of a stepped care model. For example, Renew may be useful for individuals waiting to initiate psychotherapy or who have completed psychotherapy and might benefit from Renew as a tool to help them maintain their therapy gains.

### Conclusions

Study limitations notwithstanding, our findings indicate that Renew holds promise as a self-management tool for PTSD symptoms. The effect of Renew relative to the delayed use condition was small over 6 weeks of use. However, given the scalability and cost-effectiveness of mobile mental health apps, even tools with a small effect have potential to help address the unmet need for mental health care among individuals struggling with PTSD symptoms. While not a replacement for traditional treatment, self-management tools such as Renew may represent a sufficient level of care for a proportion of veterans struggling with PTSD symptoms and may also help veterans seek a higher level of care as needed.

## Appendix

Multimedia Appendix 1 CONSORT (Consolidated Standards of Reporting Trials)-EHEALTH checklist.

Multimedia Appendix 2 Renew verbal consent script.

## Abbreviations

ANOVA: analysis of variance
CONSORT: Consolidated Standards of Reporting Trials
DSM-5: Diagnostic and Statistical Manual of Mental Disorders, fifth edition
PCL-5: Posttraumatic Stress Disorder Checklist for DSM-5
PTSD: posttraumatic stress disorder
RA: research assistant
RCT: randomized controlled trial
SUDS: Subjective Units of Distress Scale

## References

[1] Vasterling J (2008). Posttraumatic stress disorder and health functioning in a non-treatment-seeking sample of Iraq war veterans: A prospective analysis. JRRD.

[2] Possemato K, Wade M, Andersen J, Ouimette P (2010). The impact of PTSD, depression, and substance use disorders on disease burden and health care utilization among OEF/OIF veterans. Psychological Trauma: Theory, Research, Practice, and Policy.

[3] Tanielian TL, Tanielian T, Jaycox L (2008). Invisible wounds of war: Psychological and cognitive injuries, their consequences, and services to assist recovery. (Vol. 1).

[4] Bergman HE, Przeworski A, Feeny NC (2017). Rates of Subthreshold PTSD Among U.S. Military Veterans and Service Members: A Literature Review. Mil Psychol.

[5] VA/DoD (2018). VA/DOD Clinical Practice Guideline for the Management of Posttraumatic Stress Disorder and Acute Stress Disorder: Clinician Summary. Focus (Am Psychiatr Publ).

[6] Morland LA, Greene CJ, Rosen CS, Kuhn E, Hoffman J, Sloan DM (2017). Telehealth and eHealth interventions for posttraumatic stress disorder. Curr Opin Psychol.

[7] (2021). Demographics of mobile device ownership and adoption in the United States. Pew Research Center.

[8] Sander LB, Schorndanner J, Terhorst Y, Spanhel K, Pryss R, Baumeister H, Messner E (2020). 'Help for trauma from the app stores?' A systematic review and standardised rating of apps for Post-Traumatic Stress Disorder (PTSD). Eur J Psychotraumatol.

[9] Linardon J, Cuijpers P, Carlbring P, Messer M, Fuller-Tyszkiewicz Matthew (2019). The efficacy of app-supported smartphone interventions for mental health problems: a meta-analysis of randomized controlled trials. World Psychiatry.

[10] Possemato K, Kuhn Eric, Johnson E, Hoffman JE, Owen JE, Kanuri N, De Stefano L, Brooks E (2016). Using PTSD Coach in primary care with and without clinician support: a pilot randomized controlled trial. Gen Hosp Psychiatry.

[11] Miner A, Kuhn E, Hoffman JE, Owen JE, Ruzek JI, Taylor CB (2016). Feasibility, acceptability, and potential efficacy of the PTSD Coach app: A pilot randomized controlled trial with community trauma survivors. Psychol Trauma.

[12] Kuhn E, Kanuri N, Hoffman JE, Garvert DW, Ruzek JI, Taylor CB (2017). A randomized controlled trial of a smartphone app for posttraumatic stress disorder symptoms. J Consult Clin Psychol.

[13] Roy MJ, Costanzo ME, Highland KB, Olsen C, Clayborne D, Law W (2017). An App a Day Keeps the Doctor Away: Guided Education and Training via Smartphones in Subthreshold Post Traumatic Stress Disorder. Cyberpsychol Behav Soc Netw.

[14] McLean CP, Levy HC, Miller ML, Tolin DF (2022). Exposure therapy for PTSD: A meta-analysis. Clin Psychol Rev.

[15] Borah EV, Holder N, Chen K (2017). Providers’ Use of Evidence-Based Treatments for Posttraumatic Stress Disorder: The Influence of Training, Attitudes, and Barriers in Military and Private Treatment Settings. Best Practices in Mental Health.

[16] Rosen CS, Matthieu MM, Wiltsey Stirman S, Cook JM, Landes S, Bernardy NC, Chard KM, Crowley J, Eftekhari A, Finley EP, Hamblen JL, Harik JM, Kehle-Forbes SM, Meis LA, Osei-Bonsu PE, Rodriguez AL, Ruggiero KJ, Ruzek JI, Smith BN, Trent L, Watts BV (2016). A Review of Studies on the System-Wide Implementation of Evidence-Based Psychotherapies for Posttraumatic Stress Disorder in the Veterans Health Administration. Adm Policy Ment Health.

[17] Larsen SE, Wiltsey Stirman Shannon, Smith BN, Resick PA (2016). Symptom exacerbations in trauma-focused treatments: Associations with treatment outcome and non-completion. Behav Res Ther.

[18] Lewis C, Roberts NP, Gibson S, Bisson JI (2020). Dropout from psychological therapies for post-traumatic stress disorder (PTSD) in adults: systematic review and meta-analysis. Eur J Psychotraumatol.

[19] Knaevelsrud C, Brand J, Lange A, Ruwaard J, Wagner B (2015). Web-based psychotherapy for posttraumatic stress disorder in war-traumatized Arab patients: randomized controlled trial. J Med Internet Res.

[20] Krupnick JL, Green BL, Amdur R, Alaoui A, Belouali A, Roberge E, Cueva D, Roberts M, Melnikoff E, Dutton MA (2017). An Internet-based writing intervention for PTSD in veterans: A feasibility and pilot effectiveness trial. Psychol Trauma.

[21] Litz BT, Engel CC, Bryant RA, Papa A (2007). A randomized, controlled proof-of-concept trial of an Internet-based, therapist-assisted self-management treatment for posttraumatic stress disorder. Am J Psychiatry.

[22] Spence J, Titov N, Dear BF, Johnston L, Solley K, Lorian C, Wootton B, Zou J, Schwenke G (2011). Randomized controlled trial of Internet-delivered cognitive behavioral therapy for posttraumatic stress disorder. Depress Anxiety.

[23] Lewis C, Farewell D, Groves V, Kitchiner NJ, Roberts NP, Vick T, Bisson JI (2017). Internet-based guided self-help for posttraumatic stress disorder (PTSD): Randomized controlled trial. Depress Anxiety.

[24] McLean Carmen P, Foa Edna B, Dondanville Katherine A, Haddock Christopher K, Miller Madeleine L, Rauch Sheila A M, Yarvis Jeffery S, Wright Edward C, Hall-Clark Brittany N, Fina Brooke A, Litz Brett T, Mintz Jim, Young-McCaughan Stacey, Peterson Alan L, STRONG STAR Consortium (2021). The effects of web-prolonged exposure among military personnel and veterans with posttraumatic stress disorder. Psychol Trauma.

[25] Torous J, Nicholas J, Larsen ME, Firth J, Christensen H (2018). Clinical review of user engagement with mental health smartphone apps: evidence, theory and improvements. Evid Based Ment Health.

[26] Baumel A, Muench F, Edan S, Kane JM (2019). Objective User Engagement With Mental Health Apps: Systematic Search and Panel-Based Usage Analysis. J Med Internet Res.

[27] Christensen H, Griffiths KM, Farrer L (2009). Adherence in internet interventions for anxiety and depression. J Med Internet Res.

[28] Andersson G, Cuijpers P (2009). Internet-based and other computerized psychological treatments for adult depression: a meta-analysis. Cogn Behav Ther.

[29] Mohr DC, Cuijpers P, Lehman K (2011). Supportive accountability: a model for providing human support to enhance adherence to eHealth interventions. J Med Internet Res.

[30] Tiet QQ, Duong H, Davis L, French R, Smith CL, Leyva YE, Rosen C (2019). PTSD coach mobile application with brief telephone support: A pilot study. Psychol Serv.

[31] Possemato K, Kuhn E, Johnson E, Hoffman JE, Owen JE, Kanuri N, De Stefano L, Brooks E (2016). Using PTSD Coach in primary care with and without clinician support: a pilot randomized controlled trial. Gen Hosp Psychiatry.

[32] Miller ML, Davis AC, McLean CP (2021). Development and Pilot Testing of a Trauma-Focused Cognitive-Behavioral Self-Management Mobile App for Post-traumatic Stress Symptoms. J. technol. behav. sci.

[33] Ashbaugh AR, Houle-Johnson S, Herbert C, El-Hage W, Brunet A (2016). Psychometric Validation of the English and French Versions of the Posttraumatic Stress Disorder Checklist for DSM-5 (PCL-5). PLoS One.

[34] Weathers F, Litz BT, Keane TM, Palmieri PA, Marx BP, Schnurr PP (2013). The PTSD checklist for DSM-5 (PCL-5). National Center for PTSD.

[35] (2013). Anxiety disorders. Diagnostic and statistical manual of mental disorders (5th ed.).

[36] Wortmann JH, Jordan AH, Weathers FW, Resick PA, Dondanville KA, Hall-Clark B, Foa EB, Young-McCaughan S, Yarvis JS, Hembree EA, Mintz J, Peterson AL, Litz BT (2016). Psychometric analysis of the PTSD Checklist-5 (PCL-5) among treatment-seeking military service members. Psychol Assess.

[37] Blevins CA, Weathers FW, Davis MT, Witte TK, Domino JL (2015). The Posttraumatic Stress Disorder Checklist for DSM-5 (PCL-5): Development and Initial Psychometric Evaluation. J Trauma Stress.

[38] Bovin MJ, Marx BP, Weathers FW, Gallagher MW, Rodriguez P, Schnurr PP, Keane TM (2016). Psychometric properties of the PTSD Checklist for Diagnostic and Statistical Manual of Mental Disorders-Fifth Edition (PCL-5) in veterans. Psychol Assess.

[39] Littell R, Milliken G, Stroup WW, Wolfinger RD, Schabenberger O (2006). SAS for Mixed Models. 2nd edition.

[40] (2014). SAS/STAT Output Delivery System: User's Guide. Version 9.4.

[41] Kraemer HC, Kupfer DJ (2006). Size of treatment effects and their importance to clinical research and practice. Biol Psychiatry.

[42] Feingold A (2009). Effect sizes for growth-modeling analysis for controlled clinical trials in the same metric as for classical analysis. Psychol Methods.

[43] Schueller SM, Tomasino KN, Mohr DC (2017). Integrating human support into behavioral intervention technologies: The efficiency model of support. Clinical Psychology: Science and Practice.

[44] McLean CP, Wachsman T, Morland L, Norman SB, Hooper V, Cloitre M (2022). The mental health impact of COVID-19-related stressors among treatment-seeking trauma-exposed veterans. J Trauma Stress.

[45] McLean CP, Back SE, Capone C, Morland L, Norman SB, Rauch SAM, Schnurr PP, Teng E, Acierno R (2022). The Impact of COVID-19 on Psychotherapy Participation Among Individuals With Posttraumatic Stress Disorder Enrolled in Treatment Research. J Trauma Stress.

