# Supplementary material for: The Effects of an Exposure-Based Mobile App on Symptoms of Posttraumatic Stress Disorder in Veterans: Pilot Randomized Controlled Trial
Source: JMIR Mhealth Uhealth. 2022 Nov 4;10(11):e38951. doi: 10.2196/38951 (PMC9675013; doi:10.2196/38951)
Supplement: Multimedia Appendix 2 [file mhealth_v10i11e38951_app2.docx]

**Renew Verbal Consent Script**

Hello, can I please speak with [participant name]? Hi this is Madeleine/Adrian calling from the National Center for PTSD. I am calling about the Renew research study you expressed interest in and wondering if you have around 5 minutes now to talk? [If no find another time.]

Great, you should have received an information sheet regarding the study in your email, have you seen this or had a chance to read it yet? [If no – “That’s ok, I just want you to be aware of it for your reference. It details your rights as a participant and contains contact information should you have any questions or concerns during the study.” If yes – continue with script.]

Great, I want to start by asking you two “yes” or “no” screening questions to ensure you are eligible for the study.

1. Have you been bothered by symptoms of posttraumatic stress impacted by a traumatic event, such as combat, natural disaster, physical assault, or sexual assault?

2. Do you own an Android smartphone?

If the answer to either question is “no” politely inform the participant that they are not eligible to participate in this research study.

Ok great, you are eligible for the study. I’d like to give you a brief overview of what you can expect. If you consent to this study, we will first ask you to complete an online survey as a baseline assessment. After the survey, you will be randomized to one of three study conditions and participate in a Renew orientation. You will be assigned by chance into one of three groups, 1) Renew use alone, 2) Renew use with coaching support, or 3) Delayed Renew use. In all conditions you will be instructed to use the Renew app as frequently as you’d like for a total of 6 weeks.

After the 6-week period is up, you will be asked to complete a post-use online survey and a phone interview, as well as a follow-up survey another 6 weeks post-use. Unless you are assigned to the delayed use group, in which case you will first have a 6-week waiting period, and then a pre- and post-use survey.

Compensation for this study totals at $100 for all study conditions. For those in the Renew use alone or Renew use with coaching support conditions, compensation will be given as follows: $25 for the post-use survey and $75 for the 6-week follow up. For those in the waitlist condition, they will receive $50 before the start of their 6-wee use period and $50 at post-use.

The risks associated with this study are temporary distress when answering survey questions or using certain Renew activities that are designed to help you to emotionally process your trauma.

Renew incorporates therapeutic principles of cognitive behavioral exposure therapy, which involves approaching avoided situations and revisiting trauma memories. This can be uncomfortable for many people, especially at first. However, because PTSD symptoms are maintained by avoidance, these activities can reduce symptoms of posttraumatic stress, especially with repeated use. In fact, exposure therapy is recommended as a first-line PTSD treatment. By completing Renew activities often, you may experience a decrease in your posttraumatic stress symptoms. However, we cannot guarantee any benefit.

As with every research study, there is always some risk of a breach of confidentiality. Our team has carefully designed procedures to minimize this and will make every effort to protect you and your information from unauthorized access. Participation is voluntary, and you can withdraw at any time. We expect to publish the results of this study for others to read about, but no participants will be individually identified. Federal and state laws and the federal medical law, known as the HIPAA Privacy Rule, also protect your privacy. By consenting, you provide your permission for the research team, and associated entities to use and disclose information protected by the HIPAA Privacy Rule. More information about this is available on the information sheet you received in your email.

For this study, we will be tracking how different sections of the app are used and for how long. It is important to know that we will not be collecting any of the content you put into the app. This information does not leave your device, so there is no way for it to be viewed by anyone other than you unless you decide to share it. All the survey and interview data that we collect will be de-identified so that no information could be directly linked to personal information that could then be linked to you, such as your name, email address, or telephone number. Do you have any questions about how your data will be used? [Answer any questions participant may have.]

Do you have any questions at this time, regarding the study? [Answer any remaining questions participant may have.]

If you have any questions, concerns or complaints during the study, you should contact the Protocol Director, Dr. Carmen McLean, who’s information is included on your study information sheet.

Do you consent to participate in this research study?

Great, thank you for agreeing to participate. We are excited to have you as a part of our study. Next, we need to set up a time for us to have another phone call so that I can give you a brief orientation to the app. You will have to complete an online survey before this phone call, so we want to give you time to do that. Is there a good time in the next couple of days we can schedule another phone call? [Schedule and record time for next phone call.]

Ok, sounds good. I will plan on giving you a call on [date and time] and then you will be able to start using Renew. Also, please remember to have your survey completed before then!

Do you have any other questions?

[Answer any questions]

Thank you for choosing to participate in our research study.
